# Long-term follow-up of patients with anti-cyclic citrullinated peptide antibody-positive connective tissue disease: a retrospective observational study including information on the HLA-DRB1 allele and citrullination dependency

**DOI:** 10.1186/s13075-020-02351-4

**Published:** 2020-10-19

**Authors:** Takeshi Iwasaki, Shuichiro Nakabo, Chikashi Terao, Kosaku Murakami, Ran Nakashima, Motomu Hashimoto, Yoshitaka Imura, Naoichiro Yukawa, Hajime Yoshifuji, Yasuo Miura, Kimiko Yurugi, Taira Maekawa, Myrthe A. M. van Delft, Leendert A. Trouw, Takao Fujii, Tsuneyo Mimori, Koichiro Ohmura

**Affiliations:** 1 Department of Rheumatology and Clinical Immunology, Graduate School of Medicine, Kyoto University, 54 Shogoin-Kawahara-cho, Sakyo-ku, Kyoto, Japan; 2 Laboratory for Statistical and Translational Genetics, RIKEN Center for Integrative Medical Sciences, Yokohama, Japan; 3 Clinical Research Center, Shizuoka General Hospital, Shizuoka, Japan; 4 The Department of Applied Genetics, The School of Pharmaceutical Sciences, University of Shizuoka, Shizuoka, Japan; 5 Department of Advanced Medicine for Rheumatic Diseases, Graduate School of Medicine, Kyoto University, Kyoto, Japan; 6 Department of Clinical Immunology and Rheumatology, Tazuke Kofukai Medical Research Institute, Kitano Hospital, Osaka, Japan; 7 Yukawa Clinic, Wakayama, Japan; 8 Department of Transfusion Medicine & Cell Therapy, Kyoto University Hospital, Kyoto, Japan; 9 Department of Rheumatology, Leiden University Medical Center, Leiden, The Netherlands; 10 Department of Immunohematology and Blood Transfusion, Leiden University Medical Center, Leiden, The Netherlands; 11 Department of Clinical Immunology and Rheumatology, Wakayama Medical University, Wakayama, Japan; 12 Ijinkai Takeda General Hospital, Kyoto, Japan

**Keywords:** Anti-cyclic citrullinated peptide antibody, Connective tissue disease, Rheumatoid arthritis, Shared epitope

## Abstract

**Background:**

The anti-cyclic citrullinated peptide (CCP) antibody is a diagnostic biomarker of rheumatoid arthritis (RA). However, some non-RA connective tissue disease (CTD) patients also test positive for the anti-CCP antibody and, thus, may ultimately develop RA. We retrospectively investigated whether anti-CCP-positive non-RA CTD patients developed RA and attempted to identify factors that may differentiate RA-overlapping CTD from pure CTD.

**Methods:**

In total, 842 CTD patients with a primary diagnosis that was not RA were selected from our CTD database as of December 2012. Anti-CCP antibody titers were obtained from a retrospective chart review or measured using stored sera. RA was diagnosed according to the 1987 revised American College of Rheumatology classification criteria. Thirty-three anti-CCP-positive non-RA CTD patients were retrospectively followed up for the development of RA. Bone erosions on the hands and feet were assessed by X-ray. Citrullination dependency was evaluated by an in-house ELISA, the HLA-DRB1 allele was typed, and the results obtained were then compared between RA-overlapping and non-RA anti-CCP-positive CTD patients.

**Results:**

Two out of 33 anti-CCP-positive CTD patients (6.1%) developed RA during a mean follow-up period of 8.9 years. X-rays were examined in 27 out of the 33 patients, and only one (3.7%) showed bone erosions. The frequency of the HLA-DRB1 shared epitope (SE) and anti-CCP antibody titers were both significantly higher in anti-CCP-positive RA-overlapping CTD patients than in anti-CCP-positive non-RA CTD patients, while no significant differences were observed in citrullination dependency.

**Conclusions:**

Anti-CCP-positive non-RA CTD patients rarely developed RA. HLA-DRB1 SE and anti-CCP antibody titers may facilitate the differentiation of RA-overlapping CTD from anti-CCP-positive non-RA CTD.

## Background

The anti-cyclic citrullinated peptide (CCP) antibody is a widely used diagnostic biomarker of rheumatoid arthritis (RA). Despite its high specificity, previous studies reported that 5–10% of non-RA connective tissue disease (CTD) patients tested positive for the anti-CCP antibody [1, 2]. These patients may develop RA in the future because the emergence of anti-citrullinated protein antibodies (ACPA), including the anti-CCP antibody, precedes the onset of RA [3–6]. However, limited information is currently available on the long-term outcomes of anti-CCP-positive non-RA CTD patients.

ACPA-positive RA is strongly associated with certain HLA-DRB1 alleles that carry specific amino acid sequences, the so-called shared epitope (SE) [7–11]. However, ACPA-positive healthy subjects, which account for 1–2% of the population [12, 13], do not have higher frequency of HLA-DRB1 SE [13]. Therefore, SE has potential as a genetic marker to distinguish RA from non-RA in the ACPA-positive population.

The existence of the anti-CCP antibody in non-RA patients, such as those with autoimmune hepatitis [14], tuberculosis [15], and systemic lupus erythematosus (SLE) [16], is not dependent on citrullination. The anti-CCP-positive sera of patients may also react with cyclic arginine peptides (CAP), in which the citrulline residues of CCP peptides are substituted with arginine residues.

Therefore, we retrospectively investigated whether anti-CCP antibody-positive non-RA CTD patients developed RA and clarified whether HLA-DRB1 SE and the citrullination dependency of the anti-CCP antibody are predictive factors for RA.

## Methods

### Patients and clinical information

Eight hundred and forty-two CTD patients were selected from the CTD database in our division as of December 2012. Diagnoses were based on the clinical judgments of individual physicians. Clinical judgments were based on the following classification criteria: the American College of Rheumatology (ACR) 1997 criteria [17] or the new Systemic Lupus International Collaborating Clinics (SLICC) 2012 classification criteria [18] for SLE, the ACR 2012 classification criteria [19] for primary Sjögren’s syndrome (pSS), the ACR 1980 classification criteria [20] for systemic sclerosis, the Bohan and Peter diagnostic criteria [21] for polymyositis/dermatomyositis, the Kasukawa’s criteria for mixed connective tissue disease (MCTD) [22], the Yamaguchi criteria for adult-onset Still’s disease (AOSD) [23], and the Assessment in SpondyloArthritis international Society (ASAS) classification criteria for spondyloarthritis [24]. To assess the reliability of the diagnosis, we evaluated all SLE patients by chart review and found that all patients fulfilled the ACR 1997 or SLICC 2012 criteria. Since some patients in the database were diagnosed with overlapping RA, the attending physicians of patients diagnosed with RA completed a questionnaire survey in January 2020 to confirm whether their patients fulfilled the 1987 revised American College of Rheumatology (ACR) criteria for the classification of RA [25]. In the present study, we used the 1987 ACR criteria, not the 2010 American College of Rheumatology/European League Against Rheumatism (ACR/EULAR) criteria [26], because the latter are not applicable to patients with symptoms that may be attributed to another disease, including CTD, and also include the anti-CCP antibody. X-rays of the hands and feet were taken in January 2020 to establish whether anti-CCP-positive non-RA CTD patients had developed bone erosions. Regarding anti-CCP-positive non-RA CTD patients who dropped out of the follow-up and RA-overlapped patients, we retrospectively evaluated X-rays of their hands and feet, which had been taken after the anti-CCP antibody became positive. To assess whether anti-CCP-positive CTD patients had suffered from arthritis during this time frame, the attending physicians of patients completed a questionnaire survey in September 2020. Smoking status of anti-CCP antibody-positive patients was obtained by chart review in September 2020.

The sera of patients were collected and stored with written informed consent at various time points in the follow-up period. The follow-up length of each patient was defined as the period from the day when serum was collected or the anti-CCP antibody test was ordered in the clinic to the last visit before January 2020 (see Supplementary Figure 1, Additional file 1). All data were analyzed anonymously.

### Measurement of anti-CCP antibody titers and citrullination dependency

Anti-CCP antibody titers were obtained from a retrospective chart review for 445 patients or were measured using the stored sera of 397 patients. In both cases, anti-CCP antibody titers were assessed using a second-generation enzyme-linked immunosorbent assay (ELISA) kit (MESACUP-2 test CCP; MEDICAL & BIOLOGICAL LABORATORIES, Nagoya, Japan). The reference range in this kit is less than 4.5 U/mL, with a level of 100 and higher being calculated as 100 because the upper limit measured in old cases was 100. To investigate whether the anti-CCP antibody became negative over time, we obtained the latest anti-CCP antibody titer in January 2020 from the medical records of 64 anti-CCP-positive CTD patients whose anti-CCP antibody titers had been measured.

Citrullination dependency was evaluated using an in-house ELISA at the Leiden University Medical Center, as described previously [27]. CAP, the arginine version of CCP (the citrulline residues of CCP were converted to arginine), and CCP were coated on the same plate, and reactivities against CAP and CCP were compared. The dependency on citrullination was evaluated by subtracting absorbance values of anti-CAP from that of anti-CCP. A sample was considered to be citrullination-dependent when the anti-CCP antibody titer was higher than the cut-off (25 arbitrary U/mL) and the absorbance values at 415 nm value for CCP was ≥ 0.1 higher than that for CAP [28].

### Genotyping of the HLA-DRB1 allele

The HLA-DRB1 allele was typed using the WAKFlow system (Wakunaga Pharmaceutical, Akitakata, Japan) and the following were classified as HLA-DRB1 SE: *01:01, *01:02, *04:01, *04:04, *04:05, *04:08, *04:10, *04:13, *04:16, *10:01, *13:03, *14:02, and *14:06, as reported previously [29].

### Statistical analysis

All statistical analyses were conducted using R version 3.6.3. In comparisons of characteristics between anti-CCP-positive non-RA CTD patients and RA-overlapping CTD patients, the Mann-Whitney *U* test was used for continuous variables and Fisher’s exact test for categorical variables. The Mann-Whitney *U* test was used to analyze absorbance difference between anti-CCP and anti-CAP patients. Significant threshold was set to *p* = 0.05.

## Results

**Fig. 1:**
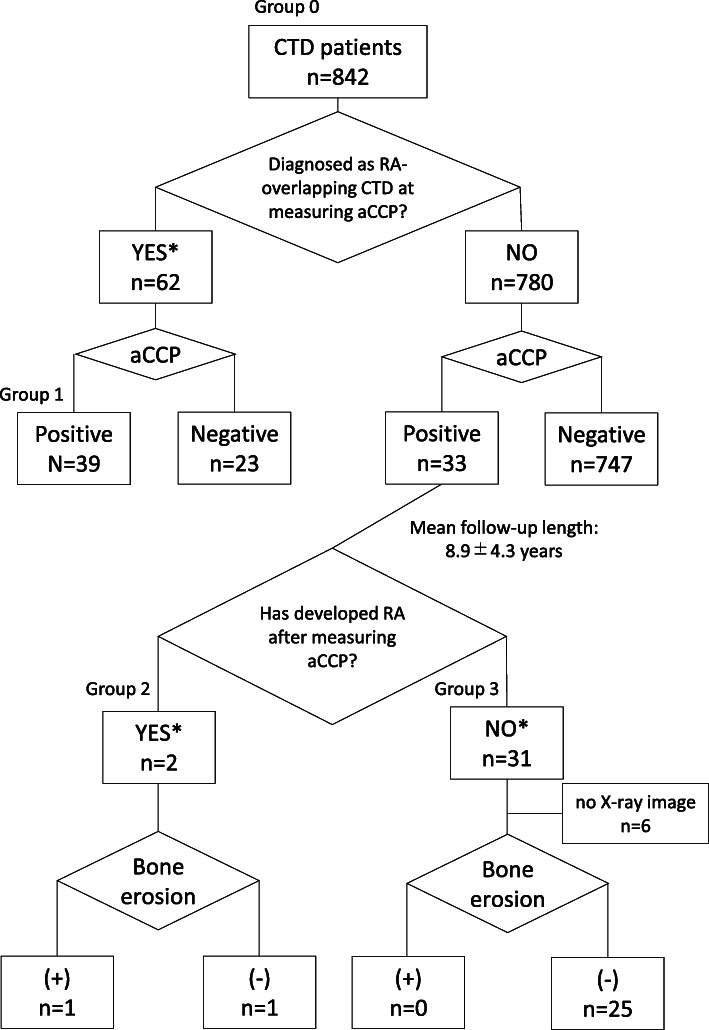
Flow chart of the classification of patients with connective tissue disease (CTD). CTD patients were classified based on the diagnosis of rheumatoid arthritis (RA), anti- cyclic citrullinated peptide (CCP) antibody positivity, and clinical characteristics. Group 1 consists of patients who fulfilled the 1987 revised American College of Rheumatology (ACR) criteria of RA before the anti-CCP antibody test was performed, group 2 consists of patients who fulfilled the 1987 ACR criteria in the follow-up period, and group 3 consists of patients never fulfilled the 1987 ACR criteria. X-rays of the hands and feet were taken for all patients in groups 1 and 2, and in 25 out of 31 patients in group 3. An asterisk denotes the accuracy of the diagnosis was reconfirmed by a questionnaire completed by each attending physician, which asked whether the patient fulfilled the 1987 revised ACR criteria for the classification of RA

A flow chart of the present study is shown in Fig. 1. We screened 842 CTD patients whose primary diagnosis was not RA in our database. Anti-CCP antibody titers were measured based on the discretion of physicians or when serum or plasma was stored regardless of joint symptoms. CTD onset times were available for 64 out of 72 patients (groups 1,2, and 3 in Fig. 1), and the mean CTD duration period at anti-CCP testing was 10.1 ± 9.4 years. Sixty-two patients were diagnosed with RA-overlapping CTD before the anti-CCP test was performed. Thirty-nine out of the 62 RA-overlapping CTD patients tested positive for the anti-CCP antibody (62.9%). On the other hand, 33 out of 780 non-RA CTD patients (4.2%) tested positive for the anti-CCP antibody. The diagnoses of the 780 non-RA CTD patients and prevalence of the anti-CCP antibody in each disease are shown in Table 1. The prevalence of the anti-CCP antibody was consistent with previous findings [2, 14, 30], except for a larger number of anti-CCP-positive patients with polymyositis/dermatomyositis than in a previous study conducted in a Western country [2].

**Table 1:** Enrolled non-RA CTD patients and the prevalence of the anti-CCP antibody

Disease	*n*	Anti-CCP-positive, *n* (%)
SLE	318	16 (5.0)
Primary SS	183	7 (3.8)
SSc	97	2 (2.1)
PM/DM	54	3 (5.6)
MCTD/overlap syndrome	60	3 (5)
AOSD	28	1 (3.6)
SpA	38∗	1 (2.6)
Others^†^	4	0 (0)
Total	780^‡^	33 (4.2)

*CTD* connective tissue disease,; systemic lupus erythematosus, *SS* Sjögren’s syndrome, *SSc* systemic sclerosis, *PM* polymyositis, *DM* dermatomyositis, *MCTD* mixed connective tissue disease, *AOSD* adult-onset Still’s disease, *SpA* spondyloarthritis

^†^Takayasu arteritis; *n* = 1, anti-phospholipid syndrome; *n* = 1, polymyalgia rheumatica; *n* = 1, polyarteritis nodosa; *n* = 1

^‡^Including overlap

^*^Thirteen out of 38 patients had erosive peripheral arthritis

We also investigated whether the 33 patients with anti-CCP-positive CTD subsequently developed RA by asking each attending physician to confirm their fulfillment of the 1987 revised ACR criteria over time. During the mean follow-up period of 8.9 years, only 2 out of 33 patients fulfilled the 1987 revised ACR criteria (Fig. 1). X-rays of the hands and feet were taken for 27 out of the 33 anti-CCP-positive CTD patients, and only one showed bone erosions (Fig. 1).

We compared the clinical characteristics of 41 anti-CCP-positive RA-overlapping CTD patients (groups 1 and 2 in Fig. 1) and 31 anti-CCP-positive non-RA CTD patients (group 3 in Fig. 1). Prevalence of arthritis in anti-CCP-positive non-RA CTD patients is shown in Supplementary Table 1, Additional file 2. The possession of HLA-DRB1 SE was also compared between 22 non-RA CTD patients and 32 RA-overlapping CTD patients. As shown in Table 2, the incidence of arthritis, prevalence of rheumatoid factor (RF), titer of the anti-CCP antibody, and usage of disease-modifying antirheumatic drugs (DMARDs) were all significantly lower in non-RA CTD patients. The anti-CCP antibody becoming negative over time was more frequently observed in non-RA CTD patients than in RA-overlapping CTD patients, although there was no statistical difference. Bone erosion was not observed in non-RA CTD patients but was frequently detected in RA-overlapping CTD patients (70.7%). The prevalence of HLA-DRB1 SE was significantly higher in RA-overlapping CTD patients (*p* = 0.01). The prevalence of HLA-DRB1 SE in RA-overlapping CTD and non-RA CTD patients was similar to that in ACPA-positive RA patients and healthy subjects in a previous study, respectively [11]. The odds ratio (OR) of developing RA for SE possession was 4.3 (95% CI 1.20–17.5). We also calculated the OR of developing RA for SE possession in patients with any smoking history (“past smokers” + “current smokers”, *n* = 9) to account for any confounding effects of smoking on RA. As a result, we found significant effect on developing RA (OR Inf (95% CI 0.7-Inf), *p* value 0.048). Overall, RF positivity, the possession of SE, and anti-CCP titers were higher in RA-overlapping CTD patients than in non-RA CTD patients.

**Table 2:** Comparison of characteristics between anti-CCP-positive non-RA CTD patients and RA-overlapping CTD patients

	Non-RA *n* = 31	RA-overlapping *n* = 41	*p* value	OR^#^ (95% CI)
Age, median (IQR), years	59.0 (41.5–68.0)	64.0 (52.0–69.0)	0.30	ND
Women, %	87.1	92.7	0.45	1.86 (0.29–13.7)
Duration of CTD, median (IQR), years	10.0 (6.0–16.0)	14.0 (8.0–21.5)	0.25	ND
Arthritis	18 (58%)	41 (100%)	2.9 × 10^−6^	Inf (5.9 Inf)
Bone erosion	0/25 (0%)	29/41 (70.7%)	1.4 × 10^−9^	Inf (12.9 Inf)
RF(+ve/−ve)	18/10 (64%)	40/1 (98%)	3.1 × 10^−4^	21.2 (2.7–982)
Titer of aCCP^†^, median (IQR), U/mL	29.4 (7.9–100)	72.6 (32.2–100)	0.045	ND
Negative conversion of aCCP	6/28 (21.4%)	5/36 (13.9%)	0.51	0.60 (0.13–2.7)
Usage of DMARDs	18 (58.1%)	37 (90.2%)	2.0 × 10^−3^	6.49 (1.69–31.3)
Usage of a medium dose of glucocorticoids^‡^	21 (67.7%)	18 (43.9%)	0.06	0.38 (0.12–1.09)
HLA-DRB1 SE^§^	6/22 (27.2%)	20/32 (62.5%)	0.014	4.3 (1.20–17.5)

*CTD* connective tissue disease, *OR* odds ratio, *95% CI* 95% confidence interval, *ND* no data, *Inf* infinite, *SD* standard deviation, *RF* rheumatoid factor, *aCCP* anti-CCP antibody, *DMARDs* disease-modifying antirheumatic drugs, including methotrexate, bucillamine, salazosulfapyridine, tacrolimus, cyclosporine, mizoribine, etanercept, and tocilizumab, *SE* shared epitope

^#^Calculated for categorical variables

^†^≥ 100 U/mL was calculated as 100 U/mL

^‡^≥ 15 mg/day of a prednisolone equivalent

^§^Assessed in 22 out of 31 patients in the non-RA group and 32 out of 41 patients in the RA-overlapping group

The positive predictive value (PPV) of anti-CCP antibody for erosive arthritis was calculated in each disease subset (*n* = 65, groups 1, 2, and 3 with X-ray images in Fig. 1) (Table 3). PPV was low in SLE, pSS, and polymyositis/dermatomyositis. However, a large proportion of systemic sclerosis patients with the anti-CCP antibody developed erosive arthritis.

**Table 3:** Positive predictive value (PPV) of the anti-CCP antibody for erosive arthritis in each disease

Disease name	Without erosion^‡^ *N* = 36	With erosion^‡^ *N* = 29	PPV %
SLE	17 (3)	6 (6)	26
Primary SS	6 (1)	0 (0)	0
SSc	2 (2)	13 (13)	87
PM/DM	7 (4)	0 (0)	0
MCTD/overlap syndrome	3 (1)	5 (5)	63
AOSD	0 (0)	1 (1)	100
SpA	1 (0)	1 (1)	50
Vasculitis	0 (0)	3^†^ (3)	100

^†^Microscopic polyangiitis; *n* = 1, polyarteritis nodosa; *n* = 1, Takayasu arteritis; *n* = 1

^‡^Number in parentheses stands for the number of RA-overlapped patients

**Fig. 2:**
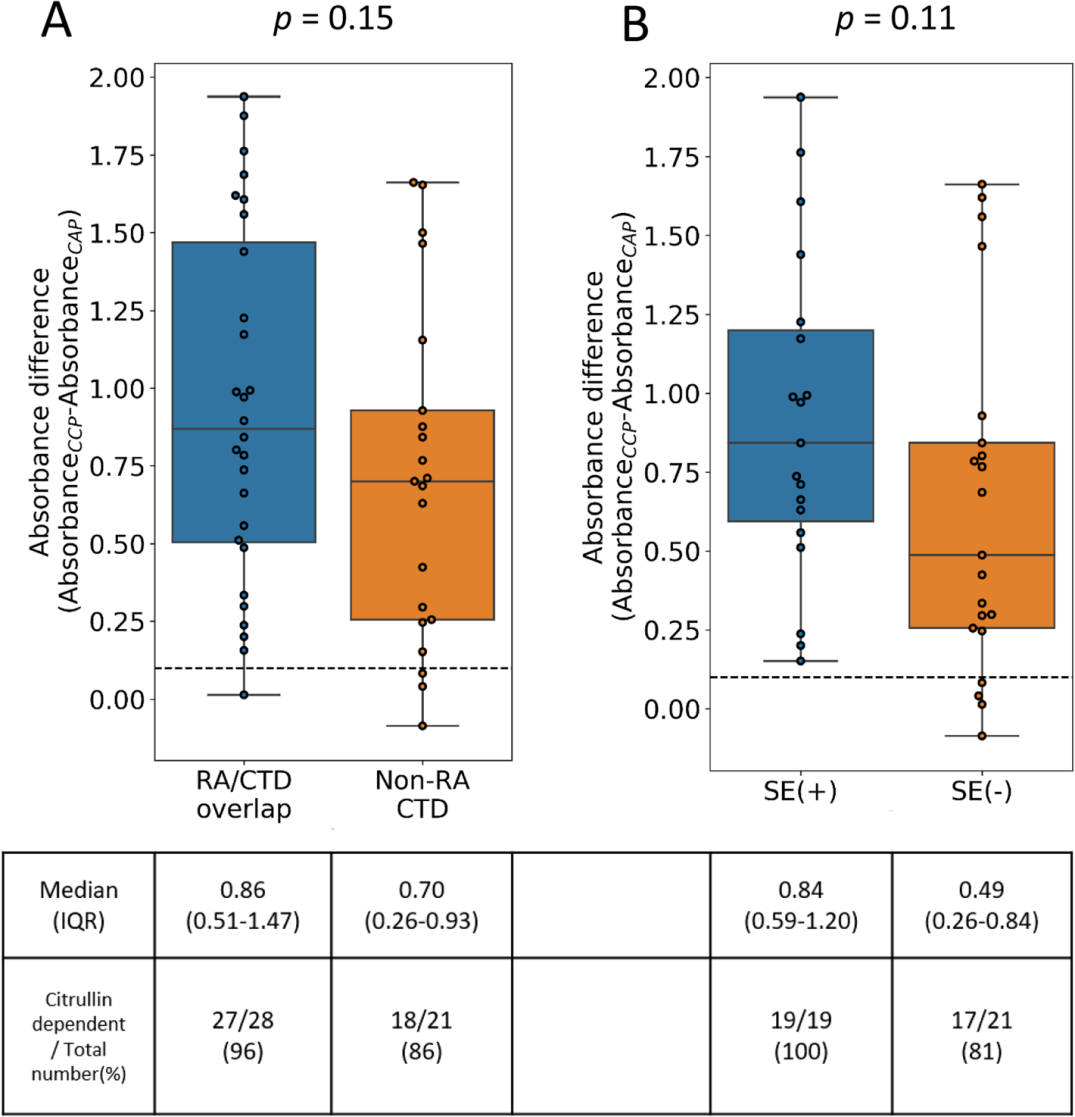
Anti-cyclic citrullinated peptide (CCP) antibody and anti-cyclic arginine peptide (CAP) antibody titers. **a** Comparison of citrullination dependency between rheumatoid arthritis (RA)-overlapping connective tissue diseases (CTD) and non-RA CTD patients. The reactivities to CAP and CCP of sera from RA-overlapping CTD patients and non-RA CTD patients were measured by an in-house ELISA and absorbance values at 415 nm, and the absorbance values of anti-CAP antibodies were subtracted from those of anti-CCP antibodies for each patient. **b** A similar analysis was performed by stratifying all samples for which HLA data were available based on positivity for the HLA-DR shared epitope (SE). Horizontal dashed lines in **a** and **b** represent the cut-off level (= 0.1) of citrullination dependency

We also investigated the citrullination dependency of anti-CCP test results because anti-CCP antibodies may react with the non-citrullinated part of CCP peptides, which have been reported in several diseases, such as SLE [16], autoimmune hepatitis [14], and tuberculosis [15]. These antibodies react with CAP, which is the arginine version of CCP (the citrulline residues of CCP were replaced by arginine). We simultaneously assessed anti-CAP and anti-CCP in 60 serum samples (33 RA-overlapping CTD and 27 non-RA CTD) and compared their reactivities. Although all 60 samples tested positive for the anti-CCP antibody using a commercial ELISA kit, 5 out of 33 RA-overlapping CTD and 6 out of 27 non-RA CTD serum samples tested negative using our in-house CCP ELISA. Therefore, we excluded anti-CCP-negative samples and examined citrullination dependency. Twenty-seven out of 28 RA-overlapping CTD patients (96%) and 18 out of 21 (85.7%) non-RA CTD patients were citrullination-dependent (Fig. 2a). There were no significant differences between the two groups (*p* = 0.15). Sensitivity/specificity of citrullination dependency for predicting RA development in the population of anti-CCP positive patients with arthritis (*n* = 39) was 96%/18%, and PPV/negative predictive value (NPV) of that was 75%/67%. In addition, we calculated the association of citrullination dependency with erosive disease. However, we could not find any significant association of citrullination dependency with erosive disease (OR = 4.4 (95% CI 0.3–244), *p* value = 0.3). We also analyzed citrullination dependency by mixing all anti-CCP-positive RA-overlapping CTD and non-RA CTD serum samples together and stratifying them by HLA-DR SE possession. Citrullination dependency was more common in patients with SE, although it was not statistically significant (*p* = 0.11) (Fig. 2b).

## Discussion

The present results revealed that anti-CCP-positive non-RA CTD patients rarely developed RA. The 1987 revised ACR criteria were used to diagnose RA; therefore, the potential effect of anti-CCP positivity on the RA or non-RA classification was excluded. Furthermore, our observation period, 8.9 years, was sufficiently long to assess the outcomes of the anti-CCP-positive population because the median period during which an anti-CCP-positive population developed RA was previously reported to be 4.5 years [4]. Ryu et al. suggested that pSS patients who test positive for the anti-CCP antibody subsequently develop RA [31]. However, the target population in the present study was pSS patients who were cross-sectionally evaluated, and RA-overlapping CTD patients were not excluded when anti-CCP antibody titers were measured. Furthermore, the 2010 ACR/EULAR criteria were used to diagnose RA [26]. Therefore, the discrepancy with the present results may be explained by differences in the target population and diagnostic criteria. One of the limitations of the present study is that information on when the anti-CCP antibody became positive was not obtained for 39 anti-CCP-positive RA-overlapping CTD patients (group 1 in Fig. 1). Since the emergence of the anti-CCP antibody may have preceded the onset of RA in these patients, anti-CCP-positive non-RA CTD patients may be more susceptible to developing RA than indicated by the present results. Despite this limitation, PPV for developing RA in non-RA CTD patients (2/33, 6.1%) was markedly lower than that in healthy individuals (82–96%) [3, 4] and similar to the incidence of RA complications in CTD patients in the present study ((group 1 and group 2)/group 0 in Fig. 1, 7.6%). Therefore, the clinical significance of the presence of the anti-CCP antibody in non-RA CTD patients remains unclear.

Limitations of our study also include the following points: (1) There is a possibility that treatment for CTD suppressed RA development in anti-CCP positive patients although there were significantly less patients who had taken DMARDs compared with RA overlapping CTD (Table 2). (2) We evaluated only anti-CCP-positive patients and did not re-evaluate the serology of anti-CCP negative CTD patients, some of whom may have newly developed anti-CCP antibody. Therefore, in this study, we cannot compare characteristics between anti-CCP positive and negative population, nor evaluate the utility of anti-CCP antibody in terms of NPV.

When we compared clinical, serological, and genetic features between non-RA CTD and RA-overlapping CTD patients, we found not only the presence of joint symptoms, bone erosion, and RF, which were all included in the 1987 revised ACR criteria [25], but also a higher anti-CCP titer and more prevalent HLA-DRB1 SE in RA-overlapping CTD patients (Table 2). Although we considered an anti-CCP titer ≥ 100 to be 100, the relationship between the anti-CCP antibody titer and the risk of developing RA is supported by patients with a high ACPA level having a high score in the 2010 ACR/EULAR classification criteria of RA [26] as well as a high anti-CCP antibody titer being more strongly associated with RA in the general population [32]. The relationship between SE and the overlap of RA in the anti-CCP-positive CTD population is consistent with previous findings showing that SE plays a crucial role in identifying which ACPA-positive patients will ultimately develop arthritis [33]. Based on these findings, a higher anti-CCP antibody titer and the presence of SE appear to be important factors in the development of RA, not only in the general population, but also in the anti-CCP-positive CTD population.

Previous studies reported that 4–10% of non-RA CTD patients tested positive for the anti-CCP antibody [1, 2], which is consistent with the present results. Due to a higher positive rate than that in the general population (1–2%) [12, 13], potential differences in autoantigens between RA-overlapping CTD patients and non-RA CTD patients may be a source of concern. In the present study, we focused on differences in the citrullination dependency of the anti-CCP antibody. Previous studies detected the anti-CCP antibody in patients with autoimmune hepatitis [34] and tuberculosis [35]; however, the epitope of this antibody was not the citrulline residue and sera reacted with the arginine version of CCP, namely, CAP [14, 15]. We speculated that the anti-CCP antibody in non-RA CTD patients was not “genuine” ACPA, but a citrullination-independent antibody. However, we found citrullination dependency was not associated with the risk of developing RA. This might be partly due to strict criterion of threshold of citrullination dependency (absorbance difference between anti-CAP and anti-CCP 0.1) for low absorbance level samples. However, absorbance difference between anti-CAP and anti-CCP was slightly larger in the RA-overlapping group (Fig. 2a). Further studies on auto-antigens of the anti-CCP antibodies in non-RA CTD patients are warranted.

## Conclusions

The present results revealed that anti-CCP-positive non-RA CTD patients rarely developed RA. RF positivity, HLA-DRB1 SE possession, and anti-CCP antibody titers may facilitate the differentiation of anti-CCP-positive RA-overlapping CTD from anti-CCP-positive non-RA CTD.

## Supplementary information

**Additional file 1: Supplementary Figure 1.** Description of the definition of the follow-up length. (PPTX 45 kb)

**Additional file 2: Supplementary Table 1.** Prevalence of arthritis in anti-CCP-positive non-RA CTD patients. (PPTX 48 kb)

## Data Availability

The datasets generated and/or analyzed in the present study are available from the corresponding author upon reasonable request.

## Abbreviations

CCP: Cyclic citrullinated peptide
RA: Rheumatoid arthritis
CTD: Connective tissue disease
SE: Shared epitope
ACPA: Anti-citrullinated protein antibodies
SLE: Systemic lupus erythematosus
ACR: American College of Rheumatology
EULAR: European League Against Rheumatism
ELISA: Enzyme-linked immunosorbent assay
CAP: Cyclic arginine peptides
OD: Optical density
DMARDs: Disease-modifying antirheumatic drugs
RF: Rheumatoid factor
PPV: Positive predictive value
NPV: Negative predictive value
pSS: Primary Sjögren’s syndrome
