# Supplementary material for: Long-term follow-up of patients with anti-cyclic citrullinated peptide antibody-positive connective tissue disease: a retrospective observational study including information on the HLA-DRB1 allele and citrullination dependency
Source: Arthritis Res Ther. 2020 Oct 19;22:248. doi: 10.1186/s13075-020-02351-4 (PMC7574466; doi:10.1186/s13075-020-02351-4)
Supplement: Supplementary file 1 — Additional file 1: Supplementary Figure 1. Description of the definition of the follow-up length. (PPTX 45 kb) [file 13075_2020_2351_MOESM1_ESM.pptx]

## Slide 1
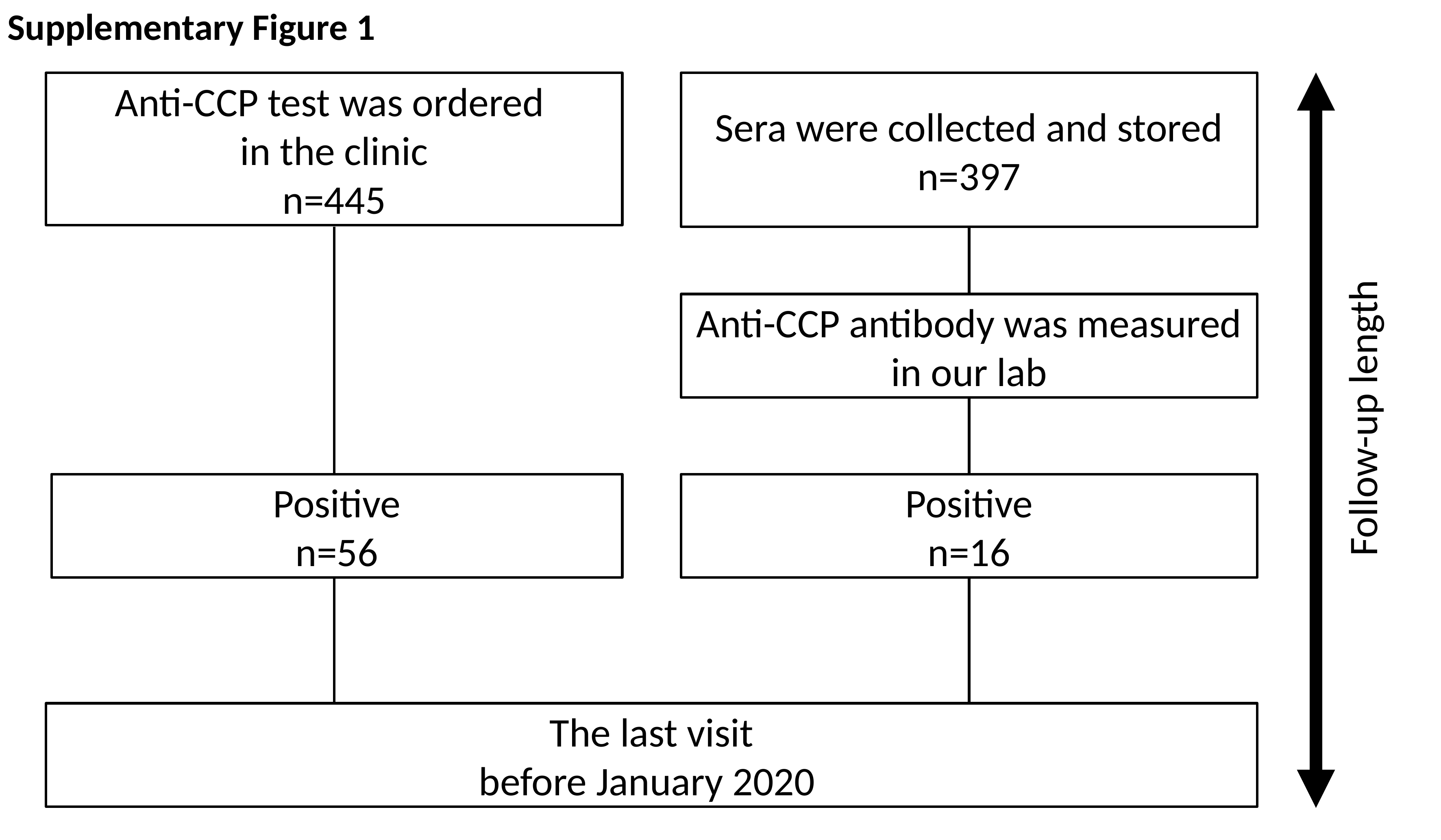

Supplementary Figure 1
Anti-CCP test was ordered
in the clinic
n=445
Sera were collected and stored
n=397
Anti-CCP antibody was measured in our lab
Follow-up length
Positive
n=56
Positive
n=16
The last visit
before January 2020
