# Supplementary figures and images for: Long-term follow-up of patients with anti-cyclic citrullinated peptide antibody-positive connective tissue disease: a retrospective observational study including information on the HLA-DRB1 allele and citrullination dependency
Source: Arthritis Res Ther. 2020 Oct 19;22:248. doi: 10.1186/s13075-020-02351-4 (PMC7574466; doi:10.1186/s13075-020-02351-4)

## Slide 1
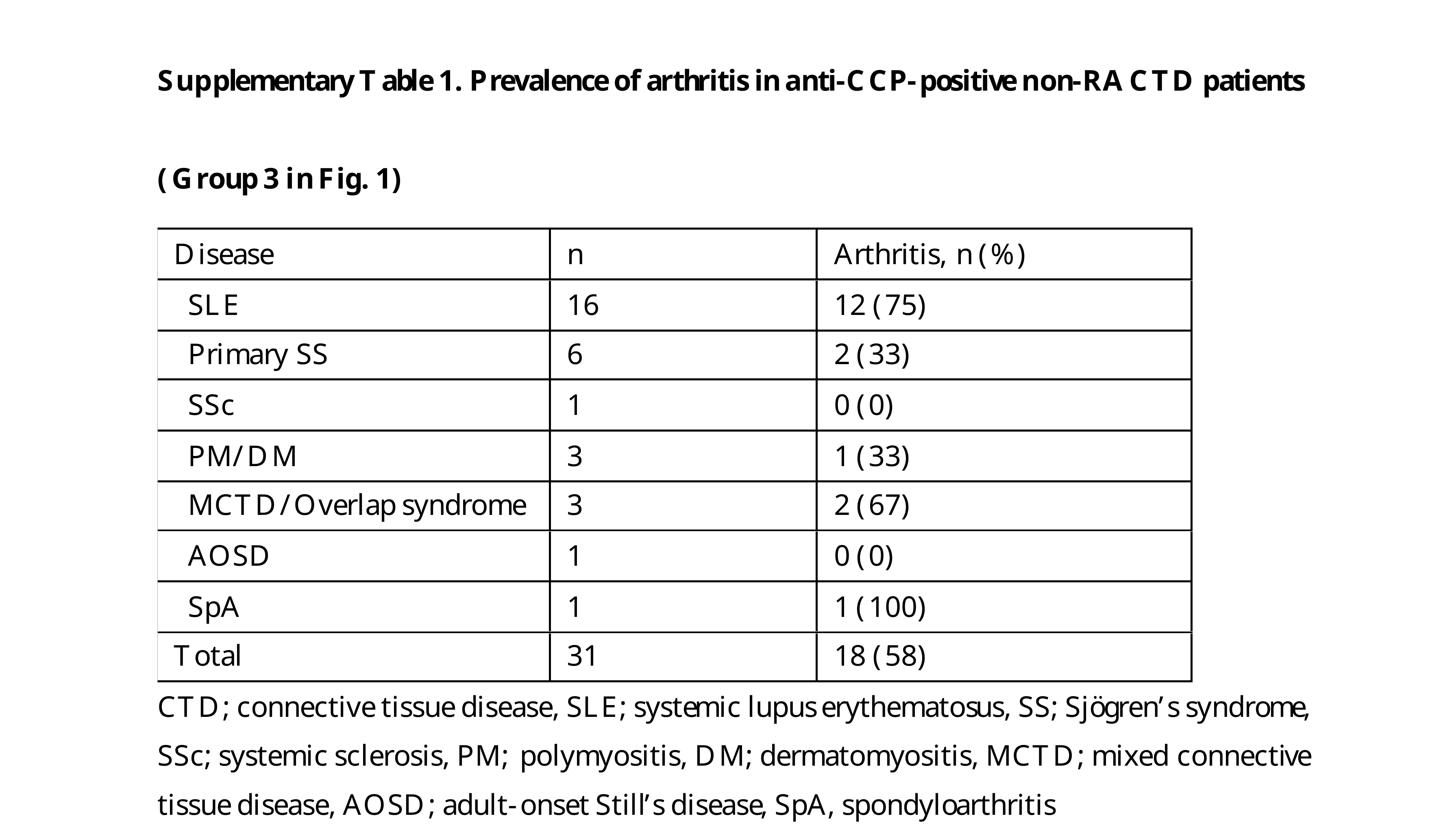

Supplement: Supplementary file 2 — Additional file 2: Supplementary Table 1. Prevalence of arthritis in anti-CCP-positive non-RA CTD patients. (PPTX 48 kb) [file 13075_2020_2351_MOESM2_ESM.pptx]
